# Intervention mapping for systematic development of a community-engaged CVD prevention intervention in ethnic and racial sexual minority men with HIV

**DOI:** 10.3389/fpubh.2025.1529152

**Published:** 2025-02-26

**Authors:** Baram Kang, Lauren Chin, Marlene Camacho-Rivera, Michael Garza, Tania de Jesús Espinosa, Xiaomei Cong, Marilyn Fraser, Mohamed Boutjdir, S. Raquel Ramos

**Affiliations:** ^1^School of Nursing, Yale University, Orange, CT, United States; ^2^Ariadne Labs, Harvard T. H. Chan School of Public Health and Brigham and Women’s Hospital, Boston, MA, United States; ^3^Department of Community Health Sciences, School of Public Health, SUNY Downstate Health Sciences University, Brooklyn, NY, United States; ^4^Arthur Ashe Institute for Urban Health, Brooklyn, NY, United States; ^5^Department of Medicine, Cell Biology and Pharmacology, State University of New York Downstate Health Sciences University, Brooklyn, NY, United States; ^6^Department of Medicine, New York University Grossman School of Medicine, New York, NY, United States; ^7^Cardiovascular Research Program, VA New York Harbor Healthcare System, Brooklyn, NY, United States; ^8^School of Public Health, Social and Behavioral Sciences, Yale University, New Haven, CT, United States; ^9^Center for Interdisciplinary Research on AIDS, Yale University, New Haven, CT, United States

**Keywords:** HIV, intervention mapping, CVD, hypertension, sexual minority men, community engagement

## Abstract

**Introduction:**

Cardiovascular disease (CVD) is a leading cause of mortality in the United States, disproportionately affecting marginalized populations such as Black and Latinx sexual minority men with HIV. These individuals face heightened CVD risk due to chronic inflammation related to HIV, side effects from treatment, and intersecting social disadvantages, including stigma and discrimination. Behavioral interventions specifically targeting these populations have been limited, with insufficient uptake in marginalized communities.

**Methods:**

This study used Intervention Mapping (IM) to develop a culturally tailored CVD prevention intervention for Black and Latinx sexual minority men with HIV. IM is a systematic, theory- and evidence-based framework for health promotion program planning. We focused on the first three of six steps in the IM process: (1) assessing community needs through literature review, framework development, and community-engaged research; (2) identifying program outcomes to develop a logic model of change; and (3) selecting theory-based methods and practical strategies for program design.

**Results:**

The needs assessment revealed significant barriers to cardiovascular health, including medical distrust, stigma, and lack of access to culturally appropriate healthcare. The logic model of change highlighted behavioral and environmental determinants influencing cardiovascular health, leading to specific performance objectives and change objectives. Strategies included leveraging eHealth technologies, such as avatar-led interactive videos, to provide private, culturally relevant health education and reduce barriers like medical distrust. Community-based participatory methods were integral to ensure the intervention was culturally resonant and acceptable.

**Discussion:**

This study demonstrated the use of IM to systematically develop a culturally tailored CVD prevention intervention for Black and Latinx sexual minority men with HIV. The findings highlight the importance of community-engaged and culturally appropriate approaches in developing interventions for historically marginalized populations. These strategies aimed to address health disparities and empower them to engage in cardiovascular health-promoting behaviors, ultimately improving cardiovascular health outcomes. Leveraging technology to foster engagement and providing culturally relevant support were crucial elements of the intervention. The insights gained may inform future cardiovascular health promotion efforts targeting similar populations.

## 1 Introduction

Cardiovascular disease (CVD) has been a leading cause of death in the United States for over 100 years (1). As the primary cause of mortality, CVD claims a life every 34 s (1). While untreated or poorly managed heart disease can result in life-altering consequences, its impact is disproportionately greater in historically marginalized communities (1).

Black and Latinx sexual minority men with HIV are more vulnerable to CVD. Compared to White and heterosexual peers, they carry a higher HIV burden (2–5). That is driven by persistent intersecting structural and social disadvantages (e.g., discrimination and stigma related to sexual identity, race, and ethnicity) (3, 4, 6, 7). This results in heightened CVD occurring from HIV-related chronic inflammation and treatment side effects (8–10). These stressors may be the catalyst for engagement in CVD risk behaviors, such as smoking, substance use, and alcohol consumption (11), that further perpetuate HIV-related CVD disparities (3).

Cardiovascular health (CVH) disparities (the differences in the prevalence, incidence, and outcomes of CVD among different demographic groups) in Black and Latinx sexual minority men are a pressing issue, and innovative prevention strategies are urgently needed (1–4, 11–14). However, behavioral CVH interventions in this population have been limited, with insufficient uptake in marginalized communities (2, 15). Extant research has not had sufficient sampling or data due to the majority of studies being less inclusive of minoritized populations (2, 15). Intervention Mapping is one advantageous approach to address this issue, as it engages community members or patients as key partners in the program planning process (16). Intervention Mapping is a rigorous, evidence-based, and reproducible approach to the development of culturally salient interventions and may be of great benefit in populations who have been historically underrepresented in research (17). The purpose of this study was to map a CVD prevention intervention for Black and Latinx sexual minority men with HIV.

## 2 Methods

Intervention Mapping is a framework for planning a theory- and evidence-based health promotion program in an iterative, stepwise process (16, 18, 19). It integrates theoretical knowledge, empirical evidence from scholarly research, and insights from the priority population (18, 19). It consists of six key steps: (1) assessing community needs to establish a logic model of the problem; (2) identifying expected program outcomes and objectives to create a logic model of change; (3) selecting theory-based methods and practical strategies to design the program; (4) producing program components; (5) planning for implementation; and (6) planning for evaluation (16, 19). Its structured, detailed protocol facilitates decision-making for program planners (16, 18, 19). In this study, we focused on the formative first three steps to describe the approach we used to develop a behavioral intervention for CVD prevention in Black and Latinx sexual minority men living with HIV.

### 2.1 Step 1: needs assessment

The first step provides the context for intervention development by assessing health-related problems and their behavioral and environmental causes. In this study, the needs assessment was based on (1) a literature review, (2) the development of a framework, and (3) interviews with the local community.

#### 2.1.1 Literature review

We conducted a scoping review of the published literature on non-pharmacological behavioral or lifestyle interventions for CVD prevention among adults living with HIV (2). The review was conducted in collaboration with a research librarian and guided by the Joanna Briggs Institute Manual for Evidence Synthesis. While our primary focus was on the prevention of hypertension—a leading CVD risk factor—we adopted a comprehensive approach by incorporating literature that explored behavioral and lifestyle strategies relevant to hypertension prevention, alongside studies that specifically targeted hypertension as an outcome. Studies that combined pharmacological management with behavioral interventions were excluded. Details of the study are found elsewhere (2).

#### 2.1.2 Framework development

Medical distrust is a longstanding obstacle to engagement in research and patient-clinician trust. This is a result of systemic racism, stigma, and discrimination to individuals based on their race, ethnicity, gender identity, and sexual orientation. Distrust of the health care system impedes access to care, adherence to medical treatment, and participation in health research. After reviewing the literature on technology-driven behavioral interventions for sexual minoritized individuals, we developed a stepwise e-Health framework that could be used to extend the reach of behavioral interventions into populations that are the most health disparate, while also acknowledging reasons for lack of trust and the need for increased privacy in empowering and non-stigmatizing ways (20).

#### 2.1.3 Local needs assessment

This research was part of a larger exploratory sequential mixed-methods study (8). We partnered with two community-based organizations in New York City that are dedicated to addressing health equity and supporting ethnic, racial, and socioeconomically minoritized populations. We conducted a local needs assessment using a community-engaged approach to develop a culturally salient CVD prevention intervention for Black and Latinx sexual minority men living with HIV. This community participatory approach is recognized as effective in enhancing the engagement of individuals who are marginalized and stigmatized due to their intersecting identities (21, 22). The needs assessment involved both quantitative and qualitative methods through survey administration, focus groups, and semi-structured interviews. Data collection using triangulated methods helps gain a comprehensive understanding of complex phenomena, especially in minoritized and underrepresented populations (23).

##### 2.1.3.1 Quantitative assessment

In the quantitative phase, validated survey measures were administered to 30 Black and Latinx sexual minority men who were members of the community-based organization. We assessed perceptions of living with chronic conditions such as HIV and comorbid hypertension and diabetes. We also assessed modifiable CVD risk behaviors, such as physical activity, tobacco, and e-cigarette use. Details about the descriptive study can be found elsewhere (24).

##### 2.1.3.2 Qualitative assessment

In the qualitative phase, we conducted focus groups with 10 HIV community experts to explore community-informed perceptions of barriers and facilitators to CVD prevention. Additionally, 30 community members who completed the survey and demographic information participated in qualitative semi-structured interviews immediately afterward. This article focuses specifically on those interviews with community members.

###### 2.1.3.2.1 Study design

We conducted semi-structured interviews using Zoom Video Communications, Inc. version 1.5, San Jose, USA, with 30 community members. The purpose of this study was to gain a deeper understanding of health concerns, HIV-related comorbid chronic conditions, and barriers and facilitators to CVD prevention. The development of this protocol was reported using the Standards for Reporting Qualitative Research (25).

###### 2.1.3.2.2 Ethics approval

The study was approved by the New York University Institutional Review Board in February 2021 (IRB-FY2021-4772) and the Yale University Institutional Review Board on May 27, 2022 (#2000031577). All procedures were in accordance with the ethical standards of the institutional and national research committee, the 1964 Helsinki Declaration and its later amendments, or comparable ethical standards. Informed consent was obtained verbally from all eligible participants who agreed to participate, given the sample characteristics of HIV diagnosis and non-heterosexual identity, as well as the minimal risk nature of the study. Each participant was compensated with a USD $45 Visa gift card as appreciation for their time.

###### 2.1.3.2.3 Participants recruitment

Recruitment strategies included word of mouth from program managers, digital flyers, and snowball sampling. Eligibility criteria were: (1) self-identifying as non-heterosexual male, (2) age 30 to 65, (3) identifying as from an ethnic or racial minoritized background, (4) HIV serostatus positive, (5) access to the internet, and (6) receiving services from a partnering community-based organization. Interested individuals who met these criteria were screened, consented, and enrolled.

###### 2.1.3.2.4 Data collection

A semi-structured interview guide included five open-ended content questions, such as “How can we improve the ways that we engage communities of color in health promotion using technology to prevent heart disease?” and “Tell me about any medical conditions other than HIV that you might be concerned about,” supplemented with probes. The interviews were conducted from May 2021 to October 2022, with each session lasting approximately 45–90 min. Participants reported that they were in a location where they felt comfortable conducting the interview. We audio-recorded every interview and assigned a pseudonym to each participant throughout the process to protect their privacy, recognizing the importance of such measures when engaging marginalized groups (21, 22). Community members were interviewed in their preferred languages, ensuring respect for their cultural values and enhancing the accuracy of the data collected (22). The principal investigator (SRR), who has extensive experience in qualitative research, conducted the interviews in English. For participants preferring to be interviewed in another language, a professional translator, approved by the community-based organizations, simultaneously interpreted the interviews in Spanish or Haitian Creole. Data saturation was achieved when no new themes emerged during the final interviews, ensuring a comprehensive exploration of participants’ experiences.

###### 2.1.3.2.5 Data analysis

Data analysis followed a five-step procedure using NVivo version 14 software (26, 27). Three authors, BK, LC, and SRR, analyzed the interview data using thematic analysis. Initially, collected data were organized and prepared for analysis. The interviews were transcribed verbatim by a certified transcription company, and the files were securely saved on a password-protected University cloud server. Subsequently, all the transcribed interviews were initially read through multiple times, with researchers taking notes to immerse themselves in the data and gain a general sense of the information. Two coders, BK and LC, then coded the data by bracketing significant words or phrases into meaning units and identifying representative categories. Half of the interview transcripts were multiply coded by both coders to enhance inter-rater reliability and consistency in coding (28). During regular meetings, authors discussed and resolved coding discrepancies, iteratively refining categories until a consensus was reached and a codebook was created. From this coding process, the authors generated themes, identifying repeatedly emerging major ideas across the transcripts. Lastly, these themes were interpreted and represented.

###### 2.1.3.2.6 Methodological rigor

Methodological rigor was ensured by adhering to four key criteria (22). First, credibility was established with a detailed interview guide and the investigator’s expertise in qualitative research methods. Credibility was further reinforced by conducting peer debriefings and member checking to ensure the data were interpreted accurately. Second, dependability was demonstrated through comprehensive descriptions of the research methods in a published study protocol (8). It was also supported by maintaining an audit trail that included a semi-structured interview guide, audio recordings, and professionally transcribed interviews. Data storage, organization, and analysis using qualitative research software facilitated stepwise replication of the study findings. Regarding data analysis, final interpretations were achieved through extensive deliberations among the three authors (BK, LC, and SRR). To ensure intercoder reliability, Cohen’s Kappa coefficient was also calculated using the NVivo version 14 software to measure the degree of agreement between coders, thereby minimizing individual biases (28). Third, transferability was supported by recruiting participants from two distinct community-based organizations. Achieving data saturation and observing consistent results across a diverse group of participants enhances the potential for the study findings to be transferable to other marginalized populations with chronic conditions. Lastly, to ensure confirmability, investigators maintained an attitude of openness to understanding participants’ lived experiences related to the intersectionality of their racial, ethnic, and sexual minoritized identities, as well as their perceptions of chronic conditions and CVD prevention, while considering their own positionality and reflexivity.

### 2.2 Step 2. Identifying expected program outcomes and objectives

Following the needs assessment, we determined the expected behavioral and environmental outcomes and determinants based on empirical literature, applicable theories, and qualitative research findings from the semi-structured interviews conducted in this study (16, 19). The expected program outcomes were differentiated into performance objectives (POs), which detailed the specific behaviors or sub-behaviors that the participants need to perform to achieve the desired outcomes (16). For each PO, the changeable determinants of behavior were selected. We also formulated change objectives (COs), which addressed changing the particular aspects of behavioral determinants so that participants are enabled to meet the POs (18). We presented the desired outcomes and determinants by creating a logic of model of change. COs of identified determinants were specified for an associated PO in a matrix of objectives.

### 2.3 Step 3. Selecting theory-based methods and practical strategies

In step 3, program developers select change methods that are grounded in theory and then choose practical strategies (16, 19). Theoretical methods refer to techniques used to achieve behavior change in line with program objectives by influencing determinants, whereas practical strategies are specific applications that deliver these methods (16, 29). The relevant theories guiding the program design were selected using three approaches: (1) reviewing previous literature on the relevant topical areas (issue approach), (2) brainstorming theoretical constructs related to interested behavior (content approach), and (3) identifying frequently used theories (general theory approach) (16). The selected theoretical methods were then implemented as practical strategies that best meet the needs of the target populations and the contexts of intervention delivery (16, 19).

## 3 Results

Systematic approaches, guided by Intervention Mapping steps, facilitated theory- and evidence-based decision-making throughout the intervention development process. We focused on the first three of six steps: (1) establishing a logic model of the problem through a needs assessment; (2) developing a logic model of change by identifying expected program outcomes and objectives; (3) selecting theory-based methods and practical strategies for program design. The findings from each of these Intervention Mapping steps are described as follows.

### 3.1 Step 1. Logic model of the problem

#### 3.1.1 Literature review

We conducted a scoping review on behavioral interventions for CVD prevention among adults living with HIV (2). It highlighted a growing emphasis on non-pharmacological, multicomponent approaches addressing lifestyle CVD risk factors, such as physical activity, diet, and weight management. Most US studies focused on the Southeast, which suggested that future research should extend to cover geographic regions that have been underrepresented and include a more comprehensive range of populations at elevated CVD risk. Details of the full review can be found elsewhere (2).

#### 3.1.2 Framework development

We have presented an innovative eHealth technology framework to shift the existing paradigm of medical distrust among sexual minority men of color in a stepwise and multi-construct approach (20). Our framework was developed in multidisciplinary collaboration with leaders in nursing, public health, and bioethics. The framework illustrates how eHealth interventions encourage engagement through the adoption and use of technology, anonymity, co-presence, self-disclosure, and social support to foster trustworthiness and trust in healthcare. We proposed the use of two eHealth modalities: (1) a virtual environment and (2) avatar-led videos (i.e., computer-generated, three-dimensional online spaces and human-like digital representations). These technologies provide private, interactive platforms that empower individuals and improve access to reliable health information, thereby promoting health behaviors in sexual minority men from racial and ethnic minority communities with chronic conditions.

#### 3.1.3 Local needs assessment

##### 3.1.3.1 Quantitative assessment

Quantitative assessment using validated survey measures revealed that most participants perceived their conditions as manageable yet serious and reported that the associated symptoms were complex. More than half did not meet the minimum recommendations for physical activity, and a third reported current nicotine use. The study findings also highlighted disparities in sleep and mental health and financial hardship associated with living with HIV. The descriptive findings of this quantitative study have been detailed elsewhere (24).

##### 3.1.3.2 Qualitative assessment

The following are the results of the qualitative data analysis.

###### 3.1.3.2.1 Participant characteristics

Among the 30 community members who participated in this study, the mean age was 47.5 years (SD = 12.5), and the mean duration since HIV diagnosis was 17.2 years (SD = 11.1, range 1–41). All participants (*N* = 30) reported having health insurance and access to care, with 97% (*n* = 29) having a regular provider and being on antiretroviral therapy. Participants reported being out of the closet for an average of 25.7 years (SD = 14.4). The majority of participants preferred the gender pronouns “he/him” (97%, *n* = 29), while one participant (3%) preferred “she/her.” For race and ethnicity, we documented their responses verbatim as participants identified themselves, adhering to the gold standard of self-identification for reporting these demographics (30). Regarding ethnic background, 70% of participants (*n* = 21) self-identified as Latinx. While Latinx ethnicity refers to having heritage from Latin America and the Caribbean, regardless of race, Haitian participants in our study did not self-identify as Latinx but Black, despite Haiti being part of Latin America. This distinction may be associated with Haiti’s unique history and culture, which are rooted in African descent, and its primary language, Haitian Creole. Further demographic information is presented in Table 1.

**Table 1:** Participant characteristics (*N* = 30).

Variables	Subcategory	*N* (%)
Race		
	Mixed or biracial	13 (43.3)
	White	9 (30)
	Haitian^a^	4 (13.3)
	Black	3 (10)
	Unspecified^b^	1 (3.3)
Ethnicity		
	Latinx^c^	21 (70)
	Non-Latinx	9 (30)
US Born		
		13 (43.3)
Highest level of academic attainment		
	High school or GED^d^	14 (46.7)
	Some college	4 (13.3)
	2- or 4-year degree	6 (20)
	Graduate degree	3 (10)
	Other	3 (10)
Employment		
	Unemployed	14 (46.7)
	Full-time	7 (23.3)
	Part-time	5 (16.7)
	Retired	3 (10)
	Disabled	1 (3.3)
Annual income		
	Less than $20,000	14 (46.7)
	$20,001–$40,000	10 (33.3)
	$40,001–$60,000	6 (20)
	$60,001 and above	0 (0)
Relationship status		
	Single	21 (70)
	Partnered	9 (30)

a As Haitian participants self-identified as Black but not Latinx, they were reported separately for race and not included in the Latinx ethnicity category.

b This participant chose not to specify their race, identifying solely as Latinx.

c Latinx ethnicity included those who racially identified as either Black or White.

d GED general educational development, equivalent to a high school diploma.

###### 3.1.3.2.2 Thematic analysis

Using inductive coding scheme, we identified nine major themes: (1) perceptions of health, (2) current and anticipated health concerns, (3) behaviors and regimens that improve health and well-being, (4) encounters with medication, (5) social encounters with in-groups and out-groups, (6) desired delivery of health education, (7) comfort in using technology and accessibility, (8) ways to nurture engagement, and (9) nurturing a safe space among users in technology-based behavioral interventions in Black and Latinx sexual minority men with HIV. Cohen’s Kappa coefficient indicated perfect intercoder agreement (*κ* = 0.95), based on Viera and Garrett’s criteria for interpreting the kappa statistic (31).

The themes and subthemes are described below, along with supporting quotes. Pseudonyms were used to safeguard the identities of participants. If a participant is referred to as “he/his/him” in the quotes below, it indicates that a translator has conveyed the participants’ words into English.

**Theme 1: Perceptions of health-**This theme focused on the overall perception of health in living with HIV. This included describing one’s health status, control over health, and perceptions of aging as subthemes.

####### 3.1.3.2.2.1 Describing one’s health status

The interviewees were asked to rate their current health. Responses ranged from unhealthy/negative through average/neutral to healthy/positive. Participants who perceived themselves as healthy described their health as “very well,” “fine,” “pretty good,” “strong and solid,” “perfect,” “super-blessed,” “completely cool,” or “free,” with some rating their health status numerically (e.g., 10 out of 10). Factors associated with positive health perceptions included regular “medical checkups,” receiving treatment and medication, not “getting sick,” not having “too many health conditions” or “any pain,” and disclosing their condition. They felt healthy when they could “work,” “be able,” and live a “normal” life, such as “going out to do [one’s] errands,” “traveling,” or “just with a little extra precaution.” Some participants evaluated their health positively when their conditions improved compared to their baseline condition or when test results, such as CD4 cell count, showed improvement.

*“I have already the treatment. I also I’m open about my condition with my friends. I do not have nothing right now that is bothering me like that. I have a good doctor. So I feel that my life is good right now, and I feel healthy.”* (Ellie, age 48).

In the average/neutral category, participants described their health status as “regular,” “fair,” “average,” “up to par,” “50–50,” and “in the middle.” Underlying conditions such as HIV and other comorbidities, uncertainty about the causes of their illness and symptoms, and the burden of taking multiple medications and dealing with their side effects prevented them from perceiving themselves as fully healthy.

*“Well, in relation to my HIV, I believe it’s really good. I mean everything is under control. But I have underlying conditions, which cause distraction in my health, so that’s why I rated myself fair.”* (Cheo, age 55).

Participants who perceived their health status as negative described managing their health as “stressful,” “very hard,” “very difficult,” and “not easy” due to HIV and comorbidities, along with a lack of “possibilities” or availability of treatment and medications. They mentioned coping mechanisms such as “denial,” ignorance, “crying,” and being “isolated” in reaction to their HIV diagnosis and reported feeling lonely, irritable, cranky, tired, depressed, and afraid.

*“Some days, I wake up being depressed. It has not been easy.”* (Yoga, age 65).

*“Because you know I have this problem with high [blood] pressure … and sometimes that I can feel a little bad for that.”* (Jesus, age 54).

####### 3.1.3.2.2.2 Control over health

The subtheme of control over health explored participants’ perceptions of how they could control their own health. Participants mentioned they could “control their own body” and “illness.” They also mentioned that their “lifestyle choices” are responsible for their health status and that it is “up to” themselves to “make well-informed decisions.” They perceived the importance of “making changes” and “taking care of [themselves]” to “manage” and “improve” their health.

*“The high blood pressure, I do believe that some like of my lifestyle choices I think is what led me to developing it. So, it is important that I kind of like have been able to manage it with like medicine and stuff.”* (Xander, age 33).

*“Your energy, your strength, and your mentality controls your illness in your body.”* (Bunny, age 32).

*“I always say; I believe HIV lives with me. I have control of what I eat, what I do to take care of myself.”* (Manuel, age 62).

####### 3.1.3.2.2.3 Perceptions of aging

Regarding aging, participants acknowledged physiological decline and reduced functionality. They mentioned experiencing or anticipating health problems they are not overly concerned about, noting that their bodies are “not like when [they] were younger.” They also discussed reduced physical activities, metabolism, and social life. Specific concerns associated with aging included physical illnesses and disabilities, such as “stiff joints” and “walking with a cane,” as well as mental issues like “loss of memory” or Alzheimer’s disease. Despite these concerns, a promising outlook on longevity while living with HIV was expressed. They believed they could still engage in health-promoting activities as they age, such as exercising at an appropriate intensity instead of “vigorous” physical activity and finding a balance between alone time and socializing.

*“Because once you grow up, you can get sick. And your health is not the same. Your body’s not the same. Your body changes.”* (Atlantic, age 47).

(Translator response) *“But you know, when you have age and your elderly, you cannot do it as much.”* (Roseman, age 62).

**Theme 2: Current and anticipated health concerns-**This theme explored the health concerns that participants were experiencing and those they worried about facing in the future. Participants expressed significant concerns about chronic, long-term health conditions. When discussing the potential sources of these concerns, they frequently referenced their family’s heredity, family medical history, and observations within their community.

####### 3.1.3.2.2.4 Current health concerns

While participants reported a variety of current health concerns, they largely expressed significant worries about chronic CVD, including diabetes, high blood pressure, high cholesterol, and heart disease. Other chronic conditions mentioned included gastrointestinal issues (e.g., cirrhosis, stomach ulcers), neurological conditions (e.g., seizure disorder), pulmonary diseases (e.g., breathing problems, asthma), auditory concerns (e.g., chronic tinnitus), and conditions possibly related to chronic inflammation (e.g., joint pain, carpal tunnel syndrome, plantar fasciitis). Participants also expressed concern about mental health conditions, such as post-traumatic stress disorder, depression, and anxiety, which they perceived as being associated with their HIV diagnosis and medication. Beyond chronic diseases, participants reported lifestyle-related health concerns such as overweight and sleep problems (e.g., difficulty falling asleep, obesity-induced sleep apnea). Infectious diseases, including influenza and SARS-CoV-2 infection (COVID-19), were also mentioned. Participants described these conditions as “cumbersome,” noting that they interfered with leading a normal life, including regular activities and diet. Managing these conditions often required significant lifestyle changes to meet medical recommendations and guidelines. While some participants acknowledged that their ‘lifestyle choices led [them] to developing’ these chronic conditions, others expressed uncertainty about “what’s causing what.”

*“I feel like a little depression, because you know I need to take this medicine every day for all of my life.”* (Jesus, age 54).

*“Well, my main concern is diabetes, to be honest with you. It’s one of the most challenging things that I’ve ever had to go through. It puts everything else on the backburner as far as my focus, which is on diabetes type 2. It’s really difficult to manage. You have to make drastic live-changes [sic] and diet changes.”* (Cheo, age 55).

Some participants reported having no current health concerns when their HIV-related symptoms were well controlled with medication, they had no chronic conditions or other illnesses, and their vital signs and laboratory results (e.g., blood pressure, CD4 cell counts) were well managed. They perceived themselves as free of major issues, feeling empowered to “make well-informed decisions” about their health.

####### 3.1.3.2.2.5 Future health concerns

Participants reported a range of anticipated health concerns, even though they did not exhibit related symptoms at the time. High blood pressure, diabetes, and heart attacks were highlighted as “really big problems.” They observed their immediate family members (e.g., grandparents, parents), relatives (e.g., aunts), and friends suffering from these conditions and had experienced losses as a result. Participants expressed concern about potential complications, such as diabetes-related blindness, limb loss, and limited mobility. Heart attacks were perceived as particularly serious and as conditions that could unexpectedly affect people, even young individuals in their 30s. Stroke was identified as a common health concern among transgender individuals due to the risk of blood clots as a side effect of hormonal therapy. Cancer, particularly colon cancer, was noted as a higher risk for racially and ethnically minoritized groups. Participants also worried about the exacerbation of symptoms (e.g., worsening tinnitus leading to deafness) and the sudden onset of underlying conditions (e.g., seizures), even if these were currently controlled. Additionally, there was a fear of death related to HIV and concerns about mental health issues and age-related conditions, such as memory loss, Alzheimer’s disease, stiff joints, and resulting disability. Managing these potential health issues was seen as requiring “extra effort in addition to just living with HIV and AIDS,” prompting participants to seek regular screenings and medical consultations with healthcare providers.

*“My grandmother is actually blind in one eye now due to diabetes. I’ve had some of my aunts lose limbs. … That stuff can get really serious. Diabetes is serious. People do not take it serious. It really is a serious disease. It’s more serious than they take it, to me.”* (James, age 35).

**Theme 3: Behaviors and regimens that improve health and well-being-**This theme explored the health maintenance activities that interviewees participate in or wish to adopt to maintain and improve their well-being. This encompassed physical activity, a healthy diet, medical interventions and health education, mental health support, social support, and various other activities.

####### 3.1.3.2.2.6 Promoting physical activity

When prompted to think about their physical activity, interviewees recalled activities such as “exercise,” “going to the gym more,” “walking a lot,” and “aerobic or cardio.” Physical activity levels varied due to age or comorbid health conditions. Performing physical activities was bolstered by participating in them alongside peers or incorporating them into daily routines, including daily commutes, grocery shopping, and watching television.

*“I walk a lot. I try to, if I can walk, I try not to take a bus or a train if it’s within a good walking distance about half the time. Also, I do other stuff like I kayak off the Hudson and stuff like that.”* (Jay, age 30).

*“I walk a lot and…walk with some friends or some person; I feel ready and excited, good. And when I go to the gym, I find some person I know that I can do…when I go, sincerely, when I go to the gym, I’m doing more cardio, walking or cycling, that and other activities.”* (Pedro, age 41).

####### 3.1.3.2.2.7 Dietary changes and conscious eating habits

Regarding diet, participants recounted the conscious changes they made in efforts to improve their health. Common techniques included exchanging sugar-sweetened beverages with water and limiting consumption of unhealthy and high-carbohydrate foods to “sometimes” or “one day per month.” Participants mentioned seeking information about nutrition from experts, peers, and media channels such as “the cooking channel.” Additionally, some participants mentioned how cultural background influenced their dietary decisions.

*“Before, I used to not care. And I’d eat a lot of fried stuff, and a lot of rice and pasta and all that stuff. But now everything is moderate with me.”* (Cindy, age 55).

*“If [my doctors] say to drink a lot of water, I drink a lot of water. If they say eat healthy, I’m trying to eat healthy. I eat chicken breasts, salmon, white rice, quinoa, vegetables.”* (BMW, age 63).

(Translator response) *“In the Haitian culture it’s a lot more home cooked meals than outside food. Like McDonald’s is considered junk food. McDonald’s is not…yes, we do not eat McDonald’s like that. We like home cooked meals – rice, beans, plants, and salads.”* (Eddy, age 65).

####### 3.1.3.2.2.8 Medical interventions and learning about health

This subtheme explored the ways in which community members sought to manage their health and gain information about current medications and clinical treatments to “live with HIV” and comorbid conditions. They regularly met with doctors for activities such as “to get [their] heart check on” and “colon screens” and “to follow all the things my doctor orders.” Participants consulted various sources, including professionals, such as nutritionists and therapists, and online videos. However, they expressed a specific desire to learn health information from medical providers. Learning about their HIV diagnosis and how to cope with “the virus” was described as “calming” and allowed them to feel “much better.” Participants also used preventive measures, such as vaccinations and aspirin, to protect against future illness and proactively sought after information for diseases that they could potentially encounter in the future.

(Translator response) *“He said the best answer is that you take your medication on time and you do whatever that is prescribed, like as your doctors recommend.”* (Eddy, age 65).

*“It’s like they are coming out with different medications for HIV. They came out with Descovy. They came out with so many of them. So what I do is I, sometimes, I do my research. And I look online, YouTube or videos. I really find out certain information about it. Like for me to really hear somebody, like a medical provider who knows more than we do, that would be perfect, too.”* (Rob, age 30).

####### 3.1.3.2.2.9 Mental health support

Participants navigated concerns about mental health using various techniques. Stress from HIV diagnosis and other life circumstances manifested through stress eating, panic attacks, and depression. Participants lessened their mental burden by “socializing and connecting” with peers and family who shared similar health experiences. Outside of these interpersonal relationships, they also practiced meditation, scheduled “quiet time,” and attended therapy. In pursuit of a more relaxed lifestyle, participants also reframed their thoughts, such as having their minds focus “on other things” and “not paying to attention to things that cannot affect me.”

*“So one of the things that has helped me all my life with whatever, you name it, depression or this condition or whatever, is socializing and connecting to other people that are in the same position as I am.”* (Xavier, age 39).

*“It’s always good to talk about it. The more you hold it in, the more you feel like I’m not comfortable, I do not want to express what I have. The best option I have is express your thoughts about it. Do not hold it in.”* (Rob, age 30).

####### 3.1.3.2.2.10 Social support

The subtheme of social support explored how participants leveraged their social relationships to enhance their health. Numerous participants “relied” on friends, peers living with HIV, and “positive people” to motivate their health journey in areas such as physical activity and mental wellness. These supporters offered encouraging advice such as “take 1 day at a time” and “just stay on that right path.” Their straightforward activity guidance, such as “Do the exercise. Drink a lot of water. Walk for 30 min every day,” was also beneficial in helping participants maintain their health regimens.

*“… a support group was beneficial for me. And meeting more people living with this condition helped me a lot.”* (Xavier, age 39).

*“As you do all these activities and all these actions, it makes your whole body feel better, makes you do more activities with my friends and with other people, other good role models who are there, who support me”* (Rob, age 30).

####### 3.1.3.2.2.11 Miscellaneous health and wellness practices

Participants also shared other, miscellaneous health activities they performed. They understood the detrimental impact of alcohol consumption and smoking on their health, although some admitted challenges with smoking cessation. Seeking clean air, maintaining a healthy weight, and getting sufficient sleep were seen as positive actions for well-being.

*“My asthma is always on. It always ran through my genes. But for some reason, I still smoke. And my sisters and my baby mothers and my cousins, they do not like that about me.”* (Bunny, age 32).

**Theme 4: Encounters with medication-**This theme described participants’ motivations and experiences during adherence or non-adherence to medication regimens. Challenges to medication adherence included “complicated” prescription regimens, uncomfortable side effects, and denial of HIV diagnosis.

####### 3.1.3.2.2.12 Benefits and effectiveness

Participants adhered to medications when they saw them as a path to return to a “normal life.” Preventive medications were viewed as powerful in that a regular regimen of just a single medication could prevent “drastic” health effects for HIV or other chronic conditions. Although adhering to a strict schedule was sometimes challenging, they had positive thoughts about staying on the medication.” Participants acknowledged that the progression of medication development and access had improved over time.

*“Nobody dies in this day with HIV. It’s one medication.”* (Atlantic, age 47).

*“I take even aspirins every day to prevent a stroke… I feel deep down in my heart that I’m not going stop ever taking aspirins. And I even tell my mother. She’s almost 80 years old. Take an aspirin every day. Because just with one little small pill could just prevent something so drastic. But today, honestly, I can say it’s just going just fine. Because now, those combinations of two and three pills just in one medication.”* (Cindy, age 55).

####### 3.1.3.2.2.13 Side effects and concerns

Participants described non-adherence due to deleterious side effects of medications that caused somatic symptoms such as diarrhea, acid reflux, and weight gain or resulted in psychological symptoms such as depression. The need to take several medications could also contribute to depression. When taking multiple medications at once or having a comorbid condition, participants found it challenging to determine whether discomfort stemmed from a chronic condition or the medication itself.

*“I feel super-blessed, super-blessed because I do not have to take so many pills and have different mood swings on the behalf of my medicines. One day, I was getting nauseous. Some days, I felt like I have diarrhea. And sometimes, I did not have an appetite. There was weight loss. It was very discomfort.”* (Cindy, age 55).

*“… sometimes I feel like a little depression, because you know I need to take this medicine every day for all of my life.”* (Jesus, age 54).

*“Sometimes I think I have some side effects from the medications, and like I have high blood pressure too, so that can be like, you know, some stuff that I can never really figure out like what’s causing what.”* (Jay, age 30).

**Theme 5: Social encounters with in-groups and out-groups-**This theme focused on participants’ interactions and relationships with both their peers from the community (i.e., living with HIV and having sexual, racially, and ethnically minoritized backgrounds) and individuals outside of it. Some interviewees described themselves as “a people person,” while others were more introverted. Peer relationships were usually positive, whereas interaction with out-group members varied from healing to stigmatizing.

####### 3.1.3.2.2.14 Peer interactions

Participants expressed that “meeting more people with this condition” helped them “a lot.” They also took on roles to educate and “advocate” for peers, helping them learn about HIV, chronic condition prevention (e.g., cancer), and “new information” in health.

*“Because even someone that actually was confessing to me that; how do you get this? And I explained it to them. And I like to advocate for my fellow peers, and even for myself.”* (Cindy, age 55).

####### 3.1.3.2.2.15 Interactions with others outside the community

This subtheme explored how participants navigated social interactions outside of their community. They spoke about chronic health conditions with family members or sought information from live resources. Participants noted that interactions with those outside their community could stigmatize sexual minority men with HIV due to a lack of knowledge among the general population. Some suggested that this could be resolved through greater educational outreach about HIV.

*“I’m a people person. Like if I was wanted like hardcore information and stuff, I’d be more comfortable in going to like my doctor, or like a community health center or something if they had like groups or something. Like I like to see people and hear about people’s experiences and the exceptional things, like what real people are like.”* (Xander, age 33).

*“Well with me, there’s a lot of stigma still. And this is 2022. And there’s still a stigma with HIV. In this time, people that do not inform themselves and people that are ignorant in the behalf that they try to push you to the side…A lot of my friends and fellow peers have been rejected with their family, giving them paper plates and disposable utensils, because they are family do not get informed about HIV.”* (Cindy, age 55).

*“Even on the commercials, what he sees is targeting the gay community…not just the gay community will have HIV…even the commercials sometimes stigmatizes people, because that is the connection. Everything pink. Pink, pink, pink. Even the cookies. So, it’s stereotyping.”* (Alberto, age 62).

While some participants were open about their HIV diagnosis such that “everybody” knew, others chose not to disclose their HIV status to co-workers, friends, and family due to stigma and negative judgment. They would “pretend” not to “have anything” to maintain a “normal” facade.

*“Not everybody in my circle knows because I think this is something you need to be very careful who you tell it to because of the stigma. Not because I think there’s something wrong with it per se.”* (Xavier, age 39).

**Theme 6: Desired delivery of health education-**This theme focused on the health information that participants expect to obtain and the desired approach to delivering health education in a technology-based behavioral intervention. The desired topics of health information were divided into two subthemes: (1) treatment and medication and (2) preventive and general health information. Preferred approaches, including tone, atmosphere, and methodological aspects of health education, were explored in the subtheme of health information delivery quality.

####### 3.1.3.2.2.16 Treatment and medication

Participants indicated that they wanted to learn more about HIV and current comorbid health problems, such as high blood pressure. They were particularly interested in symptom control, self-management strategies, and medication. They emphasized the importance of including updated information in the intervention (e.g., vaccination for monkeypox) and expressed a desire to obtain information on up-to-date HIV treatments and medications that are newly discovered.

*“… how to control all of the symptoms that I have, with getting through the medications.”* (BMW, age 63).

*“I want to know more information, new information that you are coming out with. That’s why I want to learn more, because it’s always good to learn.”* (Rob, age 30).

####### 3.1.3.2.2.17 Preventive and general health information

Participants expressed a desire to learn more about “preventive measures” and “what [they] can do to be better” in health, such as exercising, healthy eating, and even handling emergencies (e.g., layperson cardiopulmonary resuscitation). They wanted to know “how to avoid” the negative consequences of their health behaviors. A “decision tree” was suggested as a method to illustrate the outcomes of their actions. In addition to HIV, they were interested in learning about other conditions, including their risks, symptoms, treatability, and the types of health professionals who could serve as resources, even if these conditions were not of immediate concern.

*“I’m always trying to learn even stuff that I do not have. I do not have diabetes. I do not have high blood pressure. I do not have cancer. I do not have venereal diseases. I do not have hepatitis C. But I try to inform myself.”* (Cindy, age 55).

####### 3.1.3.2.2.18 Health information delivery quality

This subtheme examined the specific strategies and quality of health information delivery that participants desired. Participants emphasized the need for comprehensive health information, referring to it as “different stuff,” “every aspect,” and “a little bit of everything.” They also mentioned that health education should be “quick and informative,” as a “long drawn out” format causes participants to “tune out” or “check out.” Educational materials in the intervention should be simple, use easy-to-understand terminology, and include examples (e.g., how a plate should look for a healthy diet). Additionally, they expressed a preference for a positive tone, noting that pervasive negative health-related news can discourage community members. Participants highlighted the importance of reliable, well-structured sources of information, and they favored learning from a health educator who would lead group health education sessions. They envisioned the health educator as a “leader” or “navigator” who could “start a conversation” and “steer them in the right direction” during the sessions. They expected the health educator to be a “licensed” medical provider who “knows more than [they] do.”

*“In my opinion, it should not be very scientific. You know this high, scientific words, you know something simple that everybody could understand.”* (Jaime, age 61).

*“… learning more about different kinds of people, like the medical people who know about it, to teach us more information about it. That would be perfect.”* (Rob, age 30).

**Theme 7: Comfort in using technology and accessibility-**This theme focused on participants’ perceptions of using technology and their comfort levels with it. It also explored the factors influencing their access to technology.

Most participants, except for two, indicated that they were generally comfortable using technology. They described technology as “standard” these days and effective for information dissemination. Additionally, they noted that technology has been “a big help” and a “very effective way to connect with people.”

*“I’m very comfortable with technology. I love it, actually. And, I’m very comfortable making friends with people over the Internet.”* (Jesus, age 49).

However, comfort levels varied depending on the medium used, such as preferences for text messaging, specific social media platforms, or gaming. Factors influencing comfort levels also included technical accessibility and cultural acceptability. Age was largely cited as a determinant of technical accessibility. Older adults participants were often “not tech-savvy” or perceived as such by younger participants, preferring “face-to-face” communications. In contrast, younger individuals were perceived to favor “quick” online interactions or gaming. Cultural factors also played a role in accessibility, with participants mentioning that technology use can vary by race and ethnicity. Two participants expressed that they were not comfortable with technology at all due to old age, long periods of incarceration, and not having a computer at home. Nevertheless, one of these participants showed a willingness to learn and use technology.

(Translator response) *“Not a comfort. That does not apply to him. He does not have a computer at home; he’s not tech savvy. And only because he’s an elderly person, …”* (Eddy, age 65).

*“Oh well, that’s easy. Technology has been a big help. At first, I was ‘iffy’ about it because I’m really old school. I was raised by a mother that was straight up Puerto Rican from the hills of the island. But, technology kind of grows on you if you allow it to. So, in the past couple of years, I’ve been able to actually meeting in person some Facebook friends locally in the area, and you know, so I’ve made some really good friendships through technology, yes, through the Internet, and they seem to be going very well…The only barrier that I would say to something like that would be, there are a lot of people in my community, in the black and brown community, that aren’t very tech savvy. So, they really would not know how to maneuver and you know… So, I think maybe…I do not know. It’s something that is a problem, and yes…”* (Cheo, age 55).

To increase accessibility, participants emphasized the ease of use and the need for training before using general technology or specific technology-based modalities (e.g., navigating gaming interfaces). Providing “how-to videos” was suggested as a potential method to facilitate learning.

(Translator response) *“He says he would not mind, but he needs to be trained, so he’s not comfortable in doing it because he does not know how to do it. But, if someone trains me, then I would be more comfortable in doing it.”* (Roseman, age 62).

**Theme 8: Ways to nurture engagement in technology-based behavioral interventions-**This theme centered around characteristics and activities interviewees desired to see in a virtual community space for health education that would encourage their active and sustained participation.

####### 3.1.3.2.2.19 Interaction with peers

Participants desired to meet other community members through interactions that mirrored ones in real life, such as support groups and health education conversations that would be “interactive and mutual.” Additionally, participants suggested that community members could intentionally “meet new people” and “socialize” with one another by including a general profile of interests and the ability to guide other players within the virtual environment to retrieve information. In regards to introverted individuals, some interviewees were unsure of their willingness to participate, while others thought that the space would help those “not ready to come out to the world” to “connect with others and let them know that they are not alone.”

*“And they actually did not take their medication for a long time because of being in denial. But when they realize they are not alone in a video game that they can be playing by themselves at their house, it connects them with this universe of people that are feeling the same way they are. It could be helpful to them.”* (Xavier, age 39).

####### 3.1.3.2.2.20 Fun

Participants prioritized the aspect of “fun” and “games” when probed about desired activities in a virtual environment to motivate community members to uptake health information. Participants emphasized that “medical” and “learning” material could be woven into non-educational activities and should use attention-grabbing words, not boring jargon, for laypersons. Competition in a gamified setting was highlighted as a common motivator to engage and retain user participation. Some participants wanted action-oriented, “violent” activities such as “killing” or “attacking” antagonists such as “bad guys” or “heart disease” that represented the health conditions users would be trying to prevent or overcome. “Special guests,” such as drag queens, would “grab someone’s attention” and keep them “tuned in” over time.

*“This is a game so you have to keep it fun. Do not make it too…you are in school, you are doing your work and the teacher asks a question and everybody is raising their hands to see who can answer the quickest. You get home and it’s time to do homework and you do not even want to sit down and do it. You have to keep them interested; keep people…it’s not just medical, you can also put fun, regular things in here…quiz them on cars or capitals of states…small things…and get their attention. As long as you keep it fun, I feel like the healthy part can just be mixed in there, blended all in.”* (Success, age 41 and James, age 35).

*“A Monopoly game where the correct answer, throw the dice. It has to be competitive. Like I have to compete against somebody. I’m thinking about part of the game could be somewhere where people can talk. And then the rules in this house or in this club could be the games. So, I would invite people like; hey, nice to meet you. I’m Xavier. Let us talk a little bit. Hey, you like this. You like that. You know what? I challenge you to this game. So, we both get into that section on the club and start competing.”* (Xavier, age 39).

####### 3.1.3.2.2.21 Innovation

Participants expressed interest in the use of avatars due to their technological novelty and customization. They noted that avatars would “grab” their attention in a virtual space, and the virtual environment itself evoked interest since participants “did not have that in the past” to deliver information.

*“… the avatar is also good as well because a lot of the kids right now, that’s the way of what they are doing, so they can change their faces and so forth.”* (Peter, age 46).

####### 3.1.3.2.2.22 Diversity and inclusion

This subtheme included participants’ views on the current limits and desired inclusion of various languages, cultures, and ages in the behavioral interventions. They emphasized multilingual content to prevent “language barriers” and ensure that participants “understand what they are seeing.” They also desired the inclusion of “Hispanic” and “Afro” cultures, such as through the use of culturally familiar foods in diet education, so that they could more easily relate to the information given. One participant also deeply emphasized the unmet need for a support space for community members over the age of 40 years based on the lack of such spaces for this age group. Participants noted that depictions of avatars and characters within a virtual space should be “broad” and represent a wide spectrum of gender identities, body types, and clothing preferences.

*“… for example, if you are talking about, what is good to eat, in order to have a healthy life? If you tell me, ok, do not eat rice and gondolas, do not eat plantains, I know that plantains and [gandules] identify Latino people, in my opinion, identifies myself. But if you tell me, oh, it’s better for you to eat broccoli and dah, dah, dah, I say, oh, that is not Latino. Even though I know it is healthy to eat broccoli, but it’s not close to Me.”* (Jaime, age 61).

*“That’s one of my big issues. And I’m being totally honest about that. Any group support, anything; oh, you have to be under 40. You have to be between 18 and 35. And I always say; what about people over 40? We still have HIV. We still have problems.”* (Atlantic, age 47).

*“But when I say “make it broad” like really open, I’m talking about all types of things; gender, also clothing, also… Because those are expressions.”* (Xavier, age 39).

####### 3.1.3.2.2.23 Trivia

This subtheme described participants’ interest in the use of trivia-like games as a feature to facilitate health information uptake. They suggested that the implementation of “true-or-false multiple choice” and trivia games in general would encourage users to learn about health “conditions.” Trivia would also increase the depth of community members’ knowledge about their own conditions when they were unable to attain the knowledge from other information sources.

*“But they are supposed to have a trivia like that. Like okay, I have cancer. I have liver problems. It’s connecting with my HIV or whatever ailment you have. And they give you the information where you can go. And they tell you where you can go or who you can call, but that’s it.”* (Atlantic, age 47).

####### 3.1.3.2.2.24 Visualization and posting

Participants suggested several means of communication for effective health education to community members. They desired spoken content and “visual” content, such as videos and diagrams, rather than written content alone, to capture users’ attention. Brief video “series” were thought to retain attention over time. Posting “billboards,” “closed captioning,” and occasional “PSAs” (i.e., public service announcements) were also suggested to deliver health information in an obvious manner without disrupting the experience of navigating a virtual environment.

(Translator response) *“He says, one, you can do videos, and you can also give health messages on how medication improves health conditions. And also, you can post them throughout like, let us say, billboards, or commercials, stuff like that.”* (Roseman, age 62).

*“I think informational links would be like diagrams and stuff, because everything is visual right now. People aren’t going to sit there and want to read a whole bunch of, you know, stuff, because everything now is like, you know, even with social media, it’s flip, you know, flip, flip, flip. So you know, even like a three-minute video with something, you know, more like a series. Like, one day you watch a video, then the next day you watch another video that is like five-minutes long. So, that keeps people’s attention where you give them like a cliff hanger at the end so that way they will want to watch the next video.”* (Jay, age 30).

####### 3.1.3.2.2.25 User-specific engagement preferences

While most participants suggested their preferred approaches, some acknowledged that engagement depends on each user’s personal interests or preferences, regardless of their ability to use the technology or its intriguing features. This means that what is available or useful to one person might not be to another.

*“It depends on the person and how frequently they are on the app as well.”* (Mr. Jean Pierre, age 50).

*“Now if you say I’ve got the most potential and you feel like I’m qualified to play for the NBA, does not mean that I want to play for the NBA. Okay?”* (Bunny, age 32).

One participant expressed a dislike of “meeting people he does not know,” even though he was comfortable with using technology and interested in behavioral health education. A few others responded that they were “not going to actually use” the program due to concerns about security and a lack of interest in the gaming format. In contrast, another opinion was that people end up using technology as a necessary tool of current trends, despite personal dislike or potential adverse effects.

*“At the end of the day, this would be a tool. … It’s like a car. A car is a tool for you to move. But if you use it wrong, you can kill somebody. So at the end of the day, people need to understand that. These are tools that you are going to use, and you decide how to use them.”* (Xavier, age 39).

**Theme 9: Nurturing a safe space among users in technology-based behavioral interventions-**Participants emphasized the need for technology-based environments to feel like safe spaces where they could choose how much personal information to share, including the option to stay anonymous. Personal privacy preferences were influenced by distrust of digital interactions due to bad actors.

####### 3.1.3.2.2.26 Privacy in virtual environments

Participants understood that privacy was valued differently among community members and that personal preferences for privacy could change over time. While some individuals were “open” and “comfortable” sharing their HIV status and “real name,” they still supported others’ needs to remain anonymous and use avatars until ready to share more about themselves in a virtual environment.

*“Well, of course privacy is very important. But, I think that if I know the decision should be made by the player. So if the player wants to use his real picture, for example, that’s ok. But if the player prefers to have an avatar, that should be ok too.”* (Jaime, age 61).

####### 3.1.3.2.2.27 Distrust and safety concerns

This subtheme explored various concerns that participants held while using online technology. They understood that individuals they met online may be “shallow” and not forthcoming with their true identity, and thus expressed caution in meeting with such individuals in real life. Another concern was the potential of a closed virtual space to be infiltrated by bad actors who did not identify as community members and who may “prey on people.” Tracking information such as cookies and unrequested follow-up messages discouraged participants from logging onto certain online websites and applications.

*“Mean for the same reason. If someone shows themselves like this person and they sustain that, and then I’m interested in meeting that person, and it comes to be that that person is not what they described. I’m describing first what can go wrong. Hmm. And even worse things could happen. Like let us meet somewhere. Of course, you need to be really careful in these types of situations. It’s a very well-known rule, even with games, technology, and apps, that you can see the person, and you are not going to meet their person in their apartment.”* (Xavier, age 39).

*“People can go online just to meet people, like even though it would be something that is around something positive, there are always those people who will try to like prey on people like that. And like somebody might join it and say yes, I’m a party of the community, and you know, learn all this information, get all the facts, just to like find somebody that they can connect and do some real craziness. Like no, maybe they are a killer, I do not know. I do not play those games”* (Xander, age 33).

*“Privacy I think it’s the main, main, #1 thing. You have to have an app with privacy. I go here. But I know when I’m finished and I close that app or whatever name is that app, they are not going to be popping up in my emails as SPAM, or whatever you call it in emails, or in my Facebook or my Twitter or whatever. I know they are not being connected.”* (Atlantic, age 47).

### 3.2 Step 2: logic model of change and matrix of objectives

**Figure 1:**
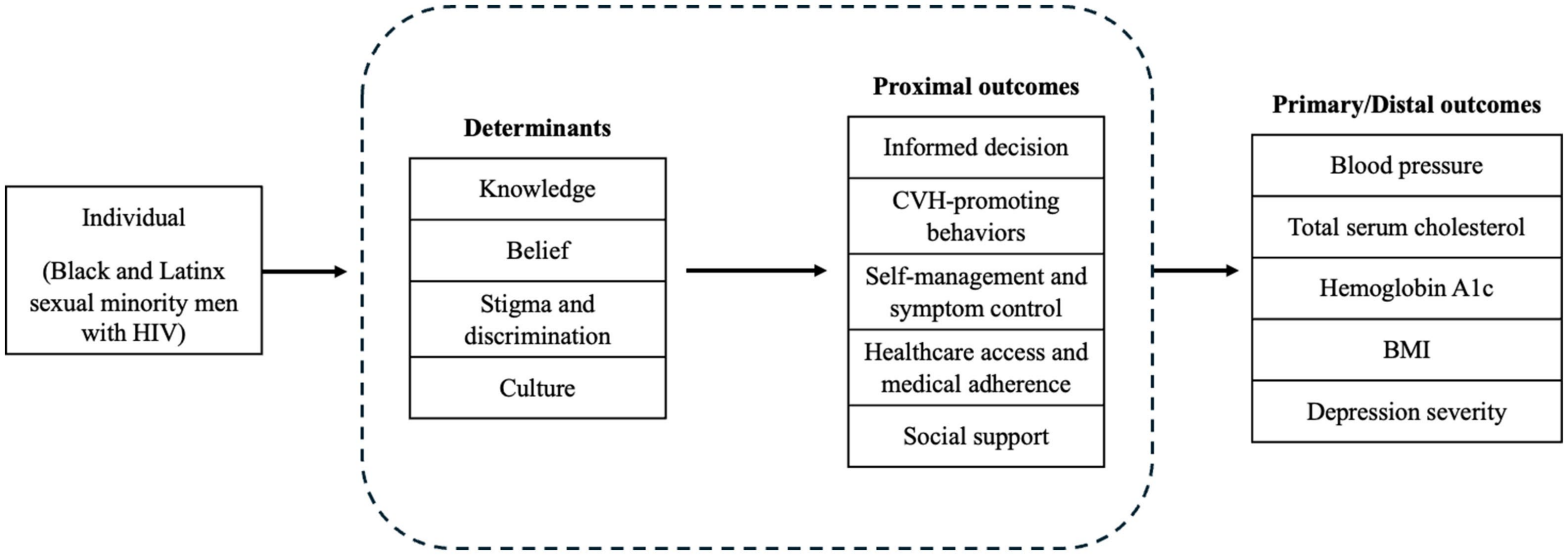
Determinants and health outcomes.

Based on the identified problems and needs in Step 1, we developed a logic model of change that outlines the expected program outcomes and their determinants (see Figure 1). In this model, outcomes are categorized into distal and proximal, reflecting the overarching goals of CVD prevention and CVH promotion through a technology-based behavioral intervention. Distal outcomes, which represent the primary goals of the intervention, include CVH-related physiological and psychological measures, such as blood pressure, total serum cholesterol, hemoglobin A1c, Body Mass Index (BMI), and depression severity. Proximal outcomes consist of specific behaviors crucial to achieving these goals: informed decision-making, CVH-promoting behaviors, self-management and symptom control, health care access and medical adherence, and social support. These proximal outcomes are directly influenced by key environmental and behavioral determinants, including knowledge, belief, medical distrust, stigma and discrimination, and culture.

To achieve the desired outcomes, we established POs at the behavioral level. For each determinant, we identified specific COs that align with the corresponding POs, detailing the actions necessary to drive these changes (see Table 2). This structured approach ensures that each determinant is addressed systematically to promote the intended health outcomes.

**Table 2:** Performance objectives and change objectives.

Performance objectives	Determinants and change objectives			
	Knowledge	Belief	Stigma and discrimination	Culture
PO 1. Understand CVH risks.	Learn about CVD risk associated with HIV and lifestyle behaviors.	Feel capable of making decisions that mitigate CVD risks based on health information.	Understand how stigma and discrimination affect CVH.	Access and comprehend health information that is culturally relevant.
PO 2. Engage in CVH-promoting behaviors.	Identify and implement health behaviors that reduce CVD risk.	Believe in their ability to implement and sustain CVH-promoting behaviors.	Implement healthy coping strategies to address stigma and discrimination-related health problems.	Choose health behaviors that align with cultural practices and community values.
PO 3. Monitor and manage symptoms effectively.	Learn how to monitor CVD-associated symptoms and what actions to take for symptom management.	Feel confident in their ability to control symptoms when they occur.	Build trust with a professional health educator and follow the guidance provided for symptom management.	Use symptom management techniques that are culturally acceptable and easily understood.
PO 4. Access and adhere to medical care.	Know the importance of regular medical check-ups and adherence to prescribed medications.	Feel responsible for having medical consultations and following medical advice.	Resolve medical distrust that may prevent access to care while interacting with a professional health educator.	Engage with healthcare providers who respect and understand cultural diversity.
PO 5. Build and maintain social support networks.	Recognize the role of social support in managing CVH.	Feel empowered to seek and maintain supportive relationships that promote health.	Have peer interactions within safe environment without judgment.	Foster social connections within a culturally diverse and respectful environment.

PO, performance objective; CVD, cardiovascular disease; CVH, cardiovascular health.

### 3.3 Step 3: theory-based methods

Diffusion of Innovations theory (32) was selected as a conceptual framework for this study. This theory explores how “new ideas, practices, and technologies” become more familiar and widely adopted within society. It encompasses five key components: (1) innovation attributes—the features of the innovation that influence its adoption; (2) adopter innovativeness—the characteristics and willingness of individuals to embrace new ideas; (3) social system and opinion leaders—the structure and influential figures who can shape attitudes and behaviors; (4) adoption process—the stages an individual goes through when adopting the innovation; and (5) diffusion system—change agency/agents and their methods of promoting the innovation within the social system (33). This theory has been frequently used in health intervention research, including studies involving sexually, racially, and ethnically minoritized men and those living with HIV (34, 35). Given that this study focused on the adoption of innovative health behaviors through a technology-based intervention for CVD prevention, the Diffusion of Innovations theory was well-suited to guide the research.

In developing this intervention, which targeted Black and Latinx sexual minority men living with HIV, we also incorporated the Social Determinants of Health Framework as applied to racial and ethnic disparities in CVD outcomes (6). This framework examines how various social, economic, and environmental factors contribute to CVH inequities, highlighting the considerable impact of structural racism and discrimination as key drivers of these disparities. Given our focus on a population from sexually minoritized and historically disadvantaged racial and ethnic communities, the Social Determinants of Health Framework provided a strong foundation for the research.

#### 3.3.1 Practical strategies

The practical strategies for this protocol were developed using the Intervention Mapping framework, emphasizing culturally tailored, digital tools like avatar-led videos and virtual environments (8). These tools were designed to address specific barriers faced by Black and Latinx sexual minority men with HIV, such as medical distrust and stigma (8). Additionally, the virtual environment behavioral intervention was premised on recommendations for CVH. The American Heart Association created Life’s Essential 8, a set of key health metrics for promoting CVH. These metrics include: (1) maintaining a heart-healthy diet, (2) engaging in physical activity (at least 150 min of moderate-intensity aerobic activity or 75 min of vigorous activity per week), (3) eliminating nicotine exposure (smoking and secondhand smoke), (4) prioritizing sleep health (7–9 h of quality sleep per night for adults), (5) achieving and maintaining a healthy body weight (BMI between 18.5–24.9), (6) managing cholesterol levels (low-density lipoprotein, high-density lipoprotein, and triglycerides), (7) controlling blood glucose (fasting blood glucose under 100 mg/dL or HbA1c less than 5.7%), and (8) maintaining optimal blood pressure (less than 120/80 mmHg) (36, 37). Recently, the American Heart Association published stroke prevention guidelines which addressed the importance of risk assessment in transgender women (37). The expansion of recommendations addressing underrepresented populations is advantageous toward inclusivity and better health for all.

When designing interventions, grounding programs in practical strategies could facilitate the uptake and adoption of heart health behaviors and ensure that health promotion is both accessible and relevant to a community’s unique cultural and social needs (18). Moreover, valuing the lived experiences of the target community, respecting and incorporating cultural values, and prioritizing the voices of the community in shaping behavioral interventions enhance the promise of achieving optimal health (20). When seeking to conduct research with ethnic and racial communities, investigators should acknowledge their social positioning, such as being someone who may or may not share the same community or lived experiences as their sample population. Acknowledging positionality is necessary to foster trust, ensure the ethical conduct of research, and make research outcomes relevant and beneficial for the communities involved (17, 22).

## 4 Discussion

The purpose of this study was to map a CVD prevention intervention for Black and Latinx sexual minority men with HIV using an iterative, evidence-based health promotion framework. Incorporating qualitative methods for local needs assessment in the Intervention Mapping approach allowed community voices to shape and tailor this informed intervention (23). The local needs assessment aimed to understand participants’ health priorities in an attempt to develop culturally salient interventions. Through qualitative semi-structured interviews, Black and Latinx sexual minority men living with HIV elucidated their specific health priorities, particularly regarding the management of HIV and CVD. These insights directly influenced the content included in the intervention, which was designed to reduce stigma, enhance engagement, and improve overall CVH outcomes.

We carefully considered the dynamics of intersecting identities when applying the Intervention Mapping framework to develop this behavioral intervention. Race, ethnicity, and sex were not viewed merely as demographic factors, but as intersectional influences that are closely linked to health behaviors and outcomes (38). By tailoring the intervention specifically for Black and Latinx sexual minority men living with HIV and incorporating an intersectional perspective, we gained a deeper understanding of their need for culturally relevant education and support. This approach allowed us to acknowledge and respect the diverse lived experiences of the participants.

We found that participants were using various online modalities as sources of health information. According to national data, the use of these modalities spans across different age and income groups. Polling data from 2023 indicated that a majority of adults aged 30 to 64 regularly used the internet (96–98%), and even 88% of adults age 65 and older reported regular internet use (39). Additionally, while participants expressed trepidations around the financial implications of using digital platforms for the purposes of conveying health literacy information, a survey conducted by Pew Research Center found that even among households earning less than $30,000 annually, 79% of individuals owned a smartphone (40). Given the ubiquity of smartphones and internet access, it has become evident that the digital divide is narrowing both in terms of accessibility and demographic use. Therefore, the opportunity for future interventions to leverage digital technologies as a means to engage with a broader range of communities will be crucial for advancing health promotion initiatives.

The National Science and Technology Council highlights the importance of fostering safety, equity, and engagement within the realm of Social-Behavioral Science (41). Our intervention promotes these values by creating an inclusive environment where Black and Latinx sexual minority men can feel safe discussing living with HIV and its associated health concerns without fear of stigma. By ensuring inclusivity and representation in the intervention design, more equitable access to health education and community engagement are promoted, ultimately leading to improved health outcomes. We observed the potential for these digital tools to enhance health promotion by leveraging a digital platform to reach participants who might otherwise be hesitant to engage in traditional face-to-face interactions. This provides both the individual users and the wider community with greater flexibility, allowing for a more convenient access point to safe, evidence-based health information. With CVD projected to increase in prevalence, especially in persons with HIV, emphasis should be placed on the critical need for innovative strategies that integrate digital tools for community-driven health promotion. Community-led initiatives are essential for achieving long-term health equity, as they enable individuals to play an active role in addressing their own health needs and priorities.

This paper focuses on the initial three steps of Intervention Mapping: (1) assessing community needs; (2) identifying expected program outcomes and objectives; and (3) selecting theory-based methods and practical strategies, which were used to describe the approach we took to develop a behavioral intervention for CVD prevention in Black and Latinx sexual minority men living with HIV. Future research is essential to explore remaining steps of Intervention Mapping: (4) producing program components; (5) planning for implementation; and (6) planning for evaluation of the intervention. These subsequent steps are crucial for understanding the intervention’s long-term impact within diverse community settings. Further investigation in these areas will contribute to the refinement of strategies aimed at promoting health equity and addressing CVD prevention in underserved populations.

### 4.1 Limitations

This study is not without limitations. First, the findings may not be generalizable to the broader population, as the sample size, although within qualitative recommendations, may not capture the full diversity of experiences and perspectives. However, we mitigated this limitation by employing measures of rigor, including a detailed interview guide, peer debriefings, and member checking, to ensure the accuracy and dependability of the data. Second, the use of Intervention Mapping as a framework for intervention development may be limited by its rigid and linear approach, which may not fully account for the complexities and nuances inherent in real-world interventions. However, we addressed this limitation by adopting a bottom-up approach, actively engaging with community members and incorporating their thoughts and perspectives into the intervention design. This collaborative approach can enhance the intervention’s effectiveness and sustainability. Third, all participants had health care access and may limit generalizability to uninsured persons. Fourth, we assessed perceptions of chronic conditions using survey measures, which carries the limitation of social desirability bias. However, our use of validated measures provided a comprehensive understanding of their perceptions, which has informed the development of this culturally salient CVD prevention intervention for Black and Latinx sexual minority men living with HIV.

## 5 Conclusion

The purpose of this study was to map a CVD prevention intervention for Black and Latinx sexual minority men with HIV using Intervention Mapping, an iterative, evidence-based health promotion framework. Qualitative methods enabled us to integrate community perspectives, shaping the culturally salient intervention tailored to our target population. Findings from this study underscore the critical need for interventions that address the intersecting identities and unique health priorities of Black and Latinx sexual minority men living with HIV. Future research should continue to prioritize community-engaged, technology-based strategies to promote CVH equity in this population.

## Data Availability

The datasets presented in this article are not readily available because the qualitative data from this study are not publicly available due to the limited sample size and the potential risk of linking contextual identifiers to participants, particularly in light of the sensitive nature of the research involving gay and bisexual men with HIV. Requests to access the datasets should be directed to Dr. S. Raquel Ramos; raquel.ramos@yale.edu.
